# Correlations between household occupancy and malaria vector biting risk in rural Tanzanian villages: implications for high-resolution spatial targeting of control interventions

**DOI:** 10.1186/s12936-016-1268-8

**Published:** 2016-04-12

**Authors:** Emmanuel W. Kaindoa, Gustav Mkandawile, Godfrey Ligamba, Louise A. Kelly-Hope, Fredros O. Okumu

**Affiliations:** Environmental Health and Ecological Sciences Thematic Group, Ifakara Health Institute, Morogoro, Tanzania; Liverpool School of Tropical Medicine, Pembroke Place, Liverpool, UK; School of Public Health, University of the Witwatersrand, Johannesburg, South Africa

**Keywords:** Malaria, Household occupancy, Targeting interventions, Hot spots, Mosquitoes

## Abstract

**Background:**

Fine-scale targeting of interventions is increasingly important where epidemiological disease profiles depict high geographical stratifications. This study verified correlations between household biomass and mosquito house-entry using experimental hut studies, and then demonstrated how geographical foci of mosquito biting risk can be readily identified based on spatial distributions of household occupancies in villages.

**Methods:**

A controlled 4 × 4 Latin square experiment was conducted in rural Tanzania, in which no, one, three or six adult male volunteers slept under intact bed nets, in experimental huts. Mosquitoes entering the huts were caught using exit interception traps on eaves and windows. Separately, monthly mosquito collections were conducted in 96 randomly selected households in three villages using CDC light traps between March-2012 and November-2013. The number of people sleeping in the houses and other household and environmental characteristics were recorded. ArcGIS 10 (ESRI-USA) spatial analyst tool, Gi* Ord Statistic was used to analyse clustering of vector densities and household occupancy.

**Results:**

The densities of all mosquito genera increased in huts with one, three or six volunteers, relative to huts with no volunteers, and direct linear correlations within tested ranges (P < 0.001). Significant geographical clustering of indoor densities of malaria vectors, *Anopheles arabiensis* and *Anopheles funestus,* but not *Culex* or *Mansonia* species occurred in locations where households with highest occupancy were also most clustered (Gi* *P* ≤ 0.05, and Gi* Z-score ≥1.96).

**Conclusions:**

This study demonstrates strong correlations between household occupancy and malaria vector densities in households, but also spatial correlations of these variables within and between villages in rural southeastern Tanzania. Fine-scale clustering of indoor densities of vectors within and between villages occurs in locations where houses with highest occupancy are also clustered. The study indicates potential for using household census data to preliminarily identify households with greatest *Anopheles* mosquito biting risk.

## Background

Significant efforts have been made to scale up appropriate interventions against malaria, an infectious tropical disease that still affects about 214 million people and kills 438,000 people annually [1]. Most of these victims are African children below 5 years old. The World Health Organization estimates that there has been a decline of malaria burden, and that morbidity worldwide reduced by 37 % and mortality by 60 % between 2000 and 2015, but sub-Saharan Africa accounts for approximately 90 % of all malaria deaths and cases [1].

In Tanzania, country-wide malaria prevalence was last estimated at 9 % among children under 5 years old, by rapid diagnostic tests (RDTs) [2]. Parasite prevalence has declined by between 50 and 60 % in most of the country since 2000, although the southeastern and northwestern parts of the country have witnessed slower gains than the rest of the country [3]. These successes are mainly attributable to scale-up of long-lasting insecticidal nets (LLINs) [4, 5] and indoor residual spraying (IRS) [6], but also improved diagnosis and treatment with effective drugs [7, 8]. It is also possible that these successes were associated with overall improved health care, improved living standards, urbanization and overall economic transformation in the country [9]. Currently, there are new efforts in the Tanzanian National Malaria Control Programme (NMCP) Strategy 2014–2020 to cut the prevalence to 5 % by 2016 and to 1 % by 2020 [10].

The current Global Technical Strategy for Malaria [11] recognizes that in order to achieve malaria elimination in today’s endemic countries, it is imperative to develop and implement not only new complementary control methods, but also improved surveillance-response strategies to support resource allocation and implementation. More emphasis is needed to develop targeted approaches in intervention campaigns focusing on residual transmission foci. The need for fine-scale targeting of interventions is growing, particularly in countries where epidemiological malaria profiles increasingly depict high geographical stratification of risk [12–14]. In many cases, as transmission levels reduce, there remains a geographically distinct pocket of transmission or demographically defined sub-populations, which must be identified and targeted to achieve zero transmission [12, 15, 16].

Based on the understanding of how disease-transmitting mosquitoes identify and follow cues from vertebrate hosts [17]. This study hypothesized that their dispersal within villages could be used as an indicator of areas where high biting risk occurs. Disease-transmitting mosquitoes are known to preferentially bite people with large body sizes [18], and households with high occupancy have also been shown to correspondingly have high *Anopheles* densities [19]. It is therefore likely that overall directional movement of mosquitoes within villages, and subsequently disease transmission risk, could be greatly influenced by spatial distribution of household biomass. In a recent study, Russel et al. demonstrate the coincidence of increased malaria transmission hazard and vulnerability occurring at the periphery of two Tanzania villages [20]. The study postulates that the occurrence of *An. gambiae* was associated with the number of occupants. The study further suggests that most vector control could be effective by targeting few households at the periphery of two villages in rural Tanzania. These observations, though widely accepted, have not previously been developed into practical actionable methodologies for disease surveillance, prevention or control. Yet this close association between human aggregations and mosquito biting risk may have significant influence on malaria parasite prevalence [21, 22] and infectiousness [23].

This study used controlled experimental hut studies and high resolution household-level sampling of indoor mosquito-biting densities to demonstrate strong spatial correlations between household occupancy and indoor malaria vector densities in three contiguous villages in south eastern Tanzania. The study also assessed whether regular household census data could be used to identify households with the greatest *Anopheles* mosquito biting risk in rural Tanzania.

## Methods

### Study area

**Fig. 1:**
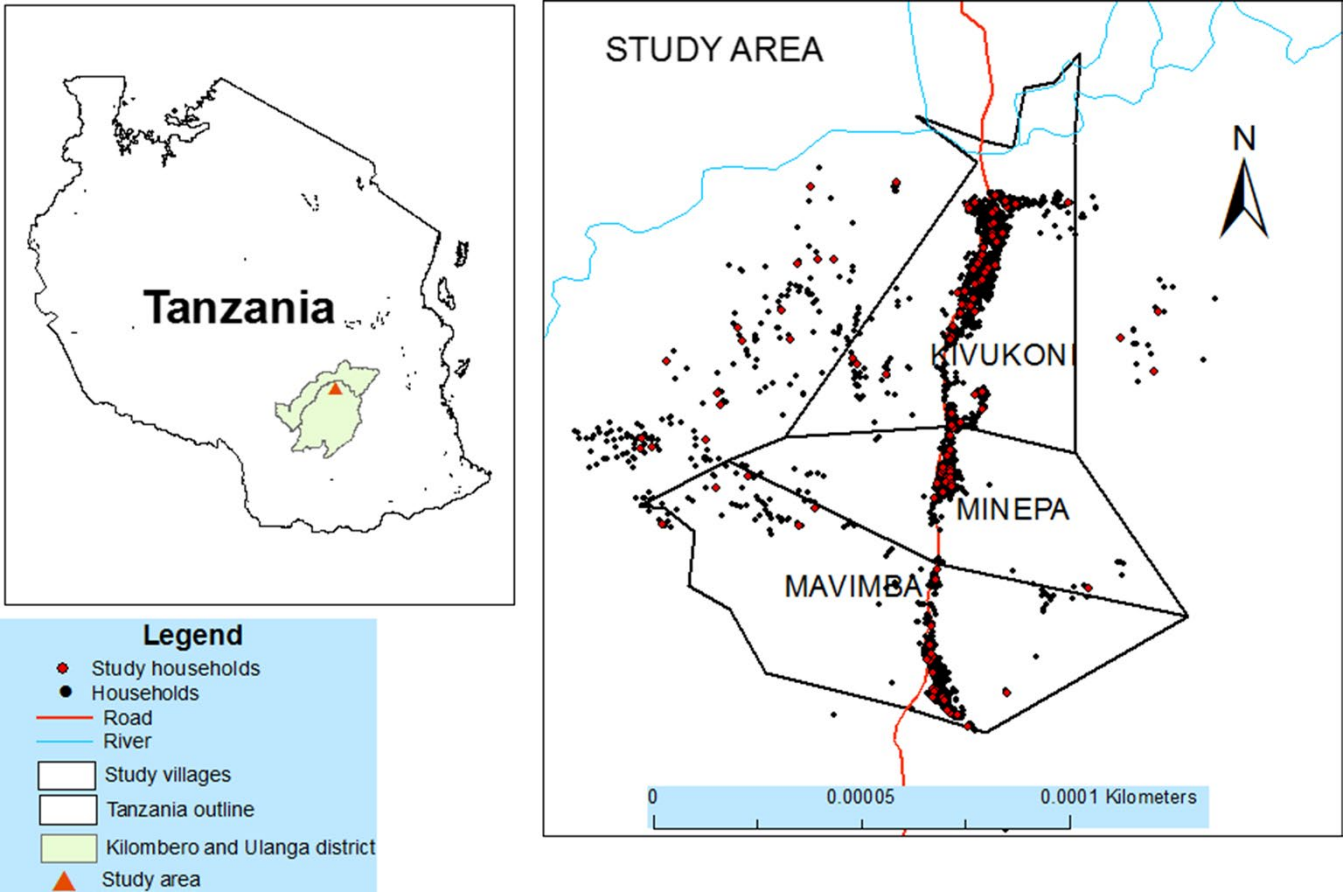
Map of the study area, showing the villages in Ulanga district where the study was conducted

The study was conducted in three villages in rural Ulanga District, southeastern Tanzania (Fig. 1). This is an area with moderate to high malaria transmission, where prevalence was last estimated at 38 % by polymerase chain reaction (PCR), 16 % by RDTs and 6 % by light microscopy [24]. Annual minimum and maximum rainfall ranges from 1200 to 1800 mm, respectively, while the mean maximum and minimum temperature are 20 and 32.6 °C, respectively. Malaria vectors in the area includes primarily *Anopheles gambiae* complex, which comprises >99 % *Anopheles arabiensis* sibling species, and *Anopheles funestus* group. Houses are mainly mud and brick walled, with thatched or iron-sheet roofs. Most people rely on subsistence farming for their livelihood, cultivating rice and maize in the Kilombero river valley.

### Study procedures

The study consisted of three parts: first, a controlled experimental assessment of effects of host biomass on mosquito house entry, using specially designed experimental huts fitted with interception exit traps on eaves and windows to collect mosquitoes that enter the huts [25]. Second, longitudinal surveys of indoor mosquito densities were conducted from March 2012 to November 2013 in randomly selected households within the Ifakara Health and Demographic Surveillance System (HDSS) area [26]. Lastly, statistical assessments and visualization of coincidental clustering between indoor malaria vector densities and household occupancy was done using ArcGIS 10 software (ESRI, USA).

### Controlled experimental verification of correlations between household occupancy and mosquito house-entry

The Ifakara experimental huts, which have previously been demonstrated as effective for studying behaviours of disease-transmitting mosquitoes, including major malaria vectors in East Africa were used [25]. The experiment was conducted in a 4 × 4 Latin square design replicated four times over 16 nights. Four experimental huts, designated A, B, C, and D were used. Each night, each of the huts was either left unoccupied, or was occupied by one, three or six volunteers. The number of volunteers for each hut was randomly assigned nightly, and was rotated across the four experimental huts over a four-night working week. A group of ten male volunteers aged between 18 and 35 years participated in the experiment throughout the 16 nights. Each night, the hut designated to have no volunteers was considered the control hut, and remained unoccupied. To eliminate any potential biases from differential attractiveness of individual volunteers to mosquitoes, the volunteers themselves randomly selected the huts in which they would sleep each night. Each day, just before the experiments began, each volunteer was asked to randomly select one piece of folded paper from a bowl containing several such folded papers, each with a specific hut label, which had been assigned by the researcher such that the specified number of volunteers per hut was always achieved. This way, there was always one hut with no volunteers (i.e. control hut); a hut with one volunteer; a hut with three volunteers; and another with six volunteers, in all cases randomly assigned. Mosquitoes were collected in interception exit traps fitted on eave spaces of the experimental huts.

### Longitudinal vector surveys to assess empirical relationships between indoor mosquito densities and household occupancy

A total of 96 households were randomly selected from an original HDSS household listing consisting of 2433 households in three villages of Kivukoni, Minepa and Mavimba, in Ulanga district, south-eastern Tanzania. The selection was conducted in two stages, where 1600 households were first selected (randomly), and spatially assigned to 16 geographical clusters each consisting of 100 households. The sampling clusters were assigned based on household latitudes so that clusters 1–16 were obtained on a north-southerly direction. From each geographical cluster, six households were selected randomly, and the household heads were requested to volunteer in the study. Whenever a household heads did not consent, the next household in the random listing was selected, so that there were always six households per cluster.

The geo-positions (latitudes and longitudes) of all the households were recorded. In the same households, the total number of people in the household, and total number of people who slept in the specific trapping room were recorded on the night of mosquito sampling. The study also observed: (a) type of roofing material (i.e. grass or iron sheets), (b) material used on walls (i.e. brick or mud), (c) whether the windows were screened or unscreened, (d) whether the eave spaces were closed or open, and (e) distance from nearest water body. All the 96 selected households were provided with a new intact long-lasting permethrin-impregnated bed net similar to what had been provided by the government during the universal LLIN coverage campaign, which covered the villages between November 2010 and January 2011 [5].

Mosquito sampling was conducted monthly in each of the study households, but the order in which the households and clusters were visited was randomized. Each week, mosquitoes were sampled in four of the 16 geographical clusters, by visiting all six households per cluster per night, working for four nights per week. In each household, one room with at least one person sleeping in it was selected for assessing indoor mosquito densities. CDC light traps set near occupied bed nets were used for the sampling [27], and were operated from 18:00 to 06:00 h. Each morning, the collected mosquitoes were killed in a closed container using petroleum fumes, then sorted by sex, taxa and physiological status as blood-fed, non-blood-fed or gravid.

A sub-sample of the female malaria vectors, *An. gambiae* complex, was examined by multiplex (PCR), which amplifies the 28S intergenic spacer region of the ribosomal DNA to distinguish between sibling species in the complex [28]. Sub-samples of *An. funestus* were also examined by PCR, using techniques developed by Koekemoer et al. [29] and Cohuet et al. [30], which are based on species specific single nucleotide polymorphisms (SNP) in the internal transcribed spacer region 2 (ITS 2). The *Anopheles* samples were also examined by enzyme-linked immunosorbent assay (ELISA), to detect *Plasmodium* sporozoites in their salivary glands [31]. To avoid false positives, all the ELISA lysates were boiled for 10 min at 100 °C, so that any detected protozoan antigens were only the heat stable *Plasmodium* species [32, 33].

### Data analysis

Data were analysed by open source software, R version 3.1.0, using the lme4 package [34]. The total number of female mosquitoes of each taxon was compared between huts having one, three or six volunteer sleepers and the control hut, being the hut with the no volunteers. The data were fitted to a generalized linear mixed effects model (GLMM), with log-linked Poisson error distribution [34]. Total mosquito catches were modelled as a function of number of volunteers and hut, while day of collection and experimental block (i.e. a set of four study nights) were used as random variables in the model, taking into account variations associated with nightly and weekly randomization in the experiment. Mean number of mosquitoes of each species collected per hut per night, and relative rates (RR) of collecting mosquitoes in the huts, and the associated 95 % Confidence intervals (CI), were estimated by exponentiating the coefficients generated from GLMMs.

For the longitudinal mosquito survey, the total number of mosquitoes collected from each house was obtained by first summing all female mosquitoes per hut per night. Relationships between household or trap room occupancy and mosquito densities were examined also by GLMMs using the *lme4* package [34], and log-linked Poisson error distributions as above. The indoor densities of mosquitoes of different species were modelled as a function of: (a) number of occupants in the mosquito trapping room, (b) total household occupancy, (c) month of mosquito collection, (d) village of collection, (e) whether the eave spaces on the houses were closed or open and (f) distance from nearest water bodies. Date of mosquito collection and house identification codes were incorporated as random variables in the GLMMs. Estimated mean indoor mosquito densities per hut per night and RR of these mosquitoes catches, and associated 95 % confidence intervals (CI), were computed from exponentials of the coefficients generated from the GLMMs.

Identification of spatial patterns of indoor mosquito catches was done using ArcGIS 10 spatial analyst tool (ESRI, USA). The Getis-Ord Gi* statistic [35, 36] in ArcGIS was used to identify locations of households with significant clustering of high indoor densities of disease-transmitting mosquitoes, including the malaria vectors, *An. arabiensis* and *An. funestus*, but also *Culex* species and *Mansonia* species. Clusters depicting both the high vector density foci (i.e. areas with households where the highest densities are most spatially concentrated) and low-vector density foci (i.e. areas with households where lowest densities are most spatially concentrated) were identified. Statistically significant clusters were then determined at a level of Gi* P value ≤0.05, and Gi* Z score ≥1.96. In this analysis, the conceptual relationship between households was assumed to be inversely related (so that houses far apart were considered more likely to be different, with regard to indoor vector densities, than households near one another), and Euclidean distances between neighbouring features were considered [35, 36].

### Ethical consideration

All human volunteer participants were fully informed of the study objectives, benefits and risks involved in the experiment. Participation was only after the volunteer provided written informed consent. All households participating in this study and all volunteers sleeping in the experimental huts were protected by intact LLINs (Olyset^®^ nets), to ensure basic minimum protection. Ethical approval for the study was obtained from Ifakara Health Institute’s Institutional Review Board (IHI/IRB/No:10-2013), Liverpool School of Tropical Medicine (Approval No. 01, issued on 10th March, 2014), and the Medical Research Coordination Committee of the National Institute of Medical Research (Certificate No. NIMR/HQ/R.8a/Vol.IX/1816). Permission to publish this manuscript was obtained from National Institute of Medical Research (Ref: NIMR/HQ/P.12 VOL XVII/16).

## Results

### Relationship between household occupancy and mosquito house-entry in experimental huts

**Fig. 2:**
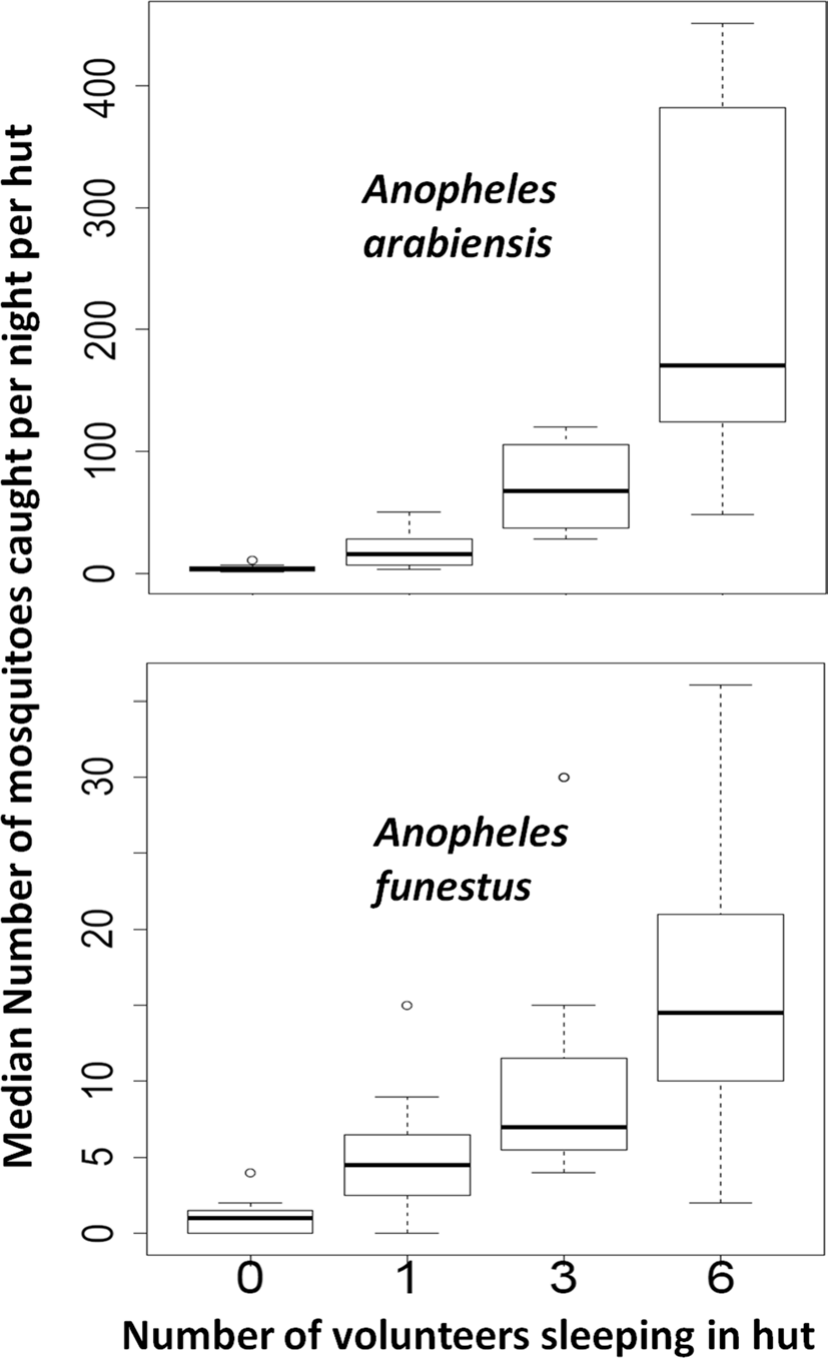
Effects of host biomass on indoor densities of malaria vectors: comparison of the number of *Anopheles arabiensis* and *Anopheles funestus* mosquitoes caught in experimental huts occupied by varying numbers of adult male volunteers. The *y-error bars* represent the inter-quartile ranges around the median

**Fig. 3:**
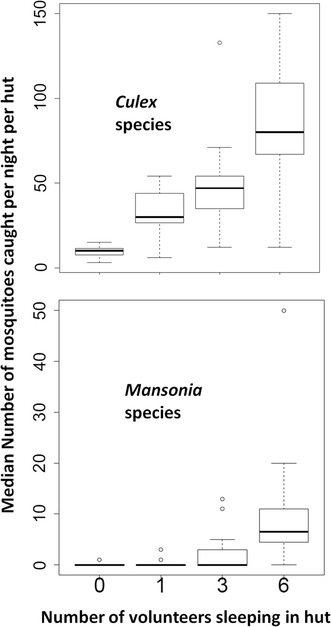
Effects of host biomass on indoor densities of non-malaria vectors: Comparison of the number of *Culex* and *Mansonia* mosquitoes caught in experimental huts occupied by varying numbers of adult male volunteers. The *y-error bars* represent the inter-quartile ranges around the median

There were significant increases in numbers of all mosquitoes of different taxa, whenever the number of volunteer sleepers (proxy of human biomass) increased in the experimental huts. The increase was observed in the catches of *An. arabiensis*, *An. funestus* and *Culex* mosquitoes. As shown in Figs. 2 and 3, the results indicate that in all huts, with one, three, or six volunteers, there were more mosquitoes than in the controls (i.e. an unoccupied hut, where occupancy is zero) (P < 0.001). This observation was valid for malaria mosquitoes, *An. arabiensis* and *An. funestus*, but also *Culex* and *Mansonia* species (Figs. 2 and 3).

### Relationships between indoor mosquito densities and household or trap room occupancy

The number of malaria vectors caught in houses increased with number of people occupying the house. To match the field observations, where trapping rooms generally had at least one person and households generally had at least two members, the study assessed effects of trap-room occupancy and household occupancies relative to base-line levels of one person versus two persons respectively. The relative rate (RR) of catching *An. arabiensis* mosquitoes in trapping rooms with more than one occupant compared to rooms with one occupant was 1.6 (95 % C.I 1.2–2.3), P < 0.001. Similarly, for *An. funestus*, trapping rooms with more than one occupant had higher catches than rooms with just one person [RR = 1.1 (1.0–1.4), P < 0.001]. A similar trend was found in densities of non-malaria mosquitoes. With regard to overall household level occupancy, as opposed to just trapping room occupancy, there were also significantly more *An. arabiensis* [RR = 1.8 (1.3–2.7), P < 0.001], more *An. funestus* [RR = 1.0 (0.9–1.2), P < 0.001] and significantly more *Culex* mosquitoes [RR = 1.3 (1.1–1.7), P < 0.001] in houses with more than two occupants, compared to houses with two or fewer occupants.

Having open eave spaces on houses was also significantly associated with indoor vector densities. The number of *An. arabiensis* caught was significantly higher in houses with open eaves, compared to houses with closed eaves [RR = 0.8 (0.4.1–1.4), P < 0.001] and the number of *An. funestus* was consistently higher in huts with open eaves compared to huts with closed eaves [RR = 1.2 (1.0–1.5) P < 0.001]. Similar trends were observed for *Culex* mosquito species [RR = 0.9 (0.7–1.1), P < 0.005], and *Mansonia* species [RR = 3.2 (2.0–5.2), P < 0.001]. In this study, the CDC light traps used for sampling mosquitoes were set near human-occupied bed nets in the selected households. Besides, all participating households were provided with ITNs and encouraged to use these nightly. It was therefore unlikely that this variable would have any effect on overall vector densities, and was excluded in the analyses.

### Spatial clustering and correlations between house occupancy and indoor vector densities

**Fig. 4:**
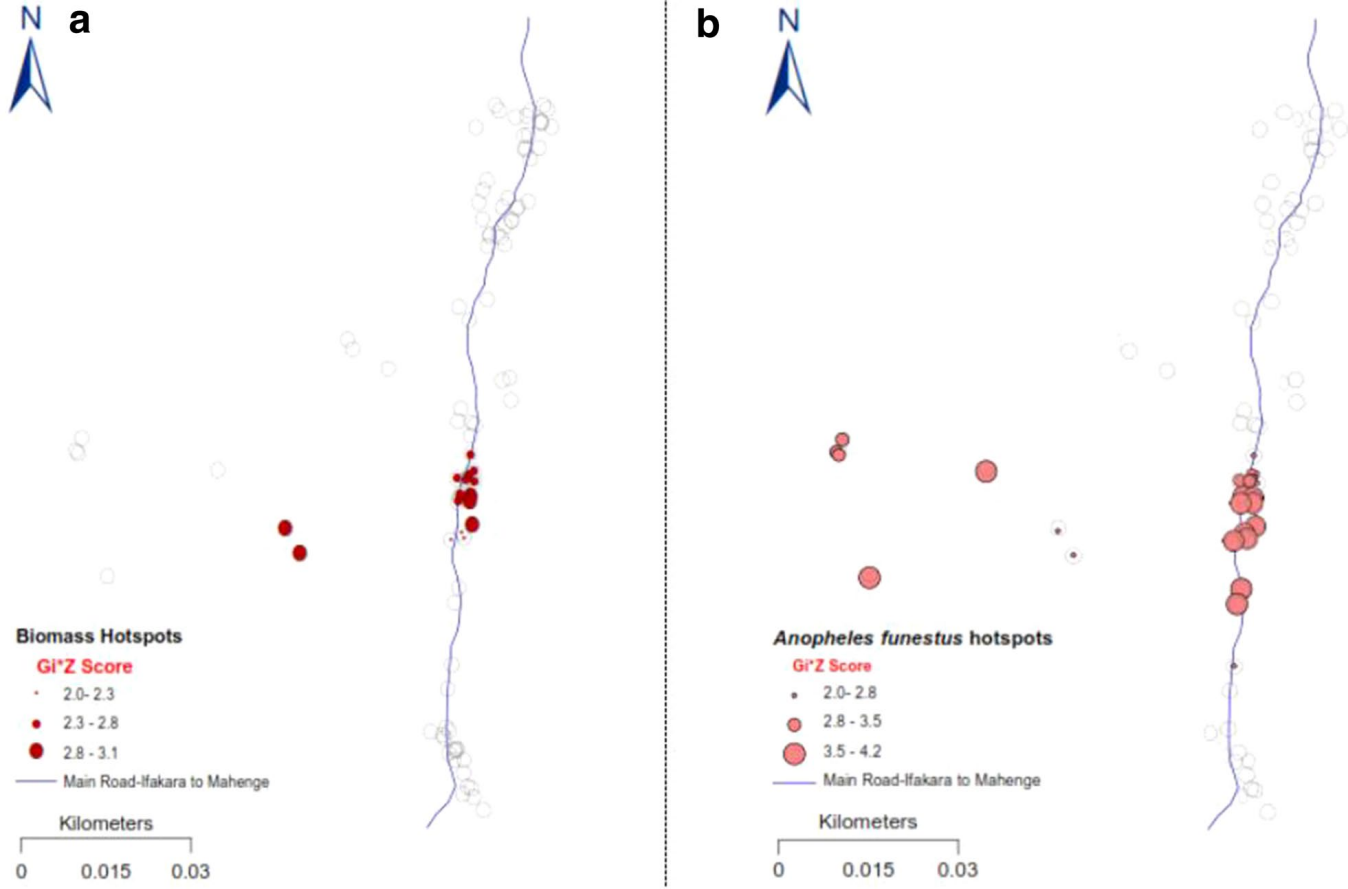
Maps showing statistically significant clusters of households with the high occupancy (**a**) and statistically significant clusters of households with high densities of *Anopheles arabiensis* in the same study area (**b**). The *grey circles* represent the rest of the households in the study area

**Fig. 5:**
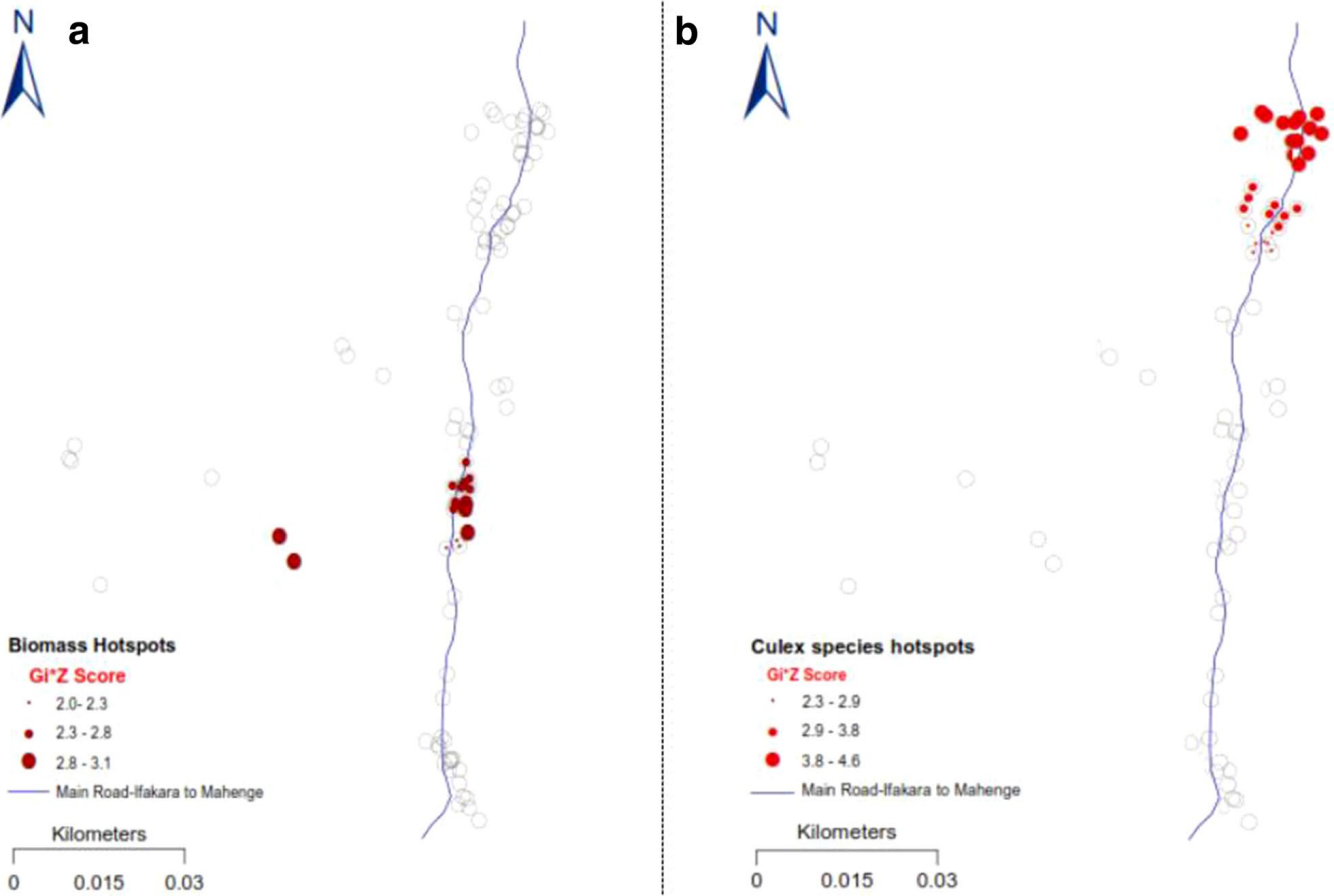
Maps showing statistically significant clusters of households with high occupancy (**a**) and statistically significant clusters of households with high densities of *Anopheles funestus* in the same study area (**b**). The *grey circles* represent the rest of the households in the study area

**Fig. 6:**
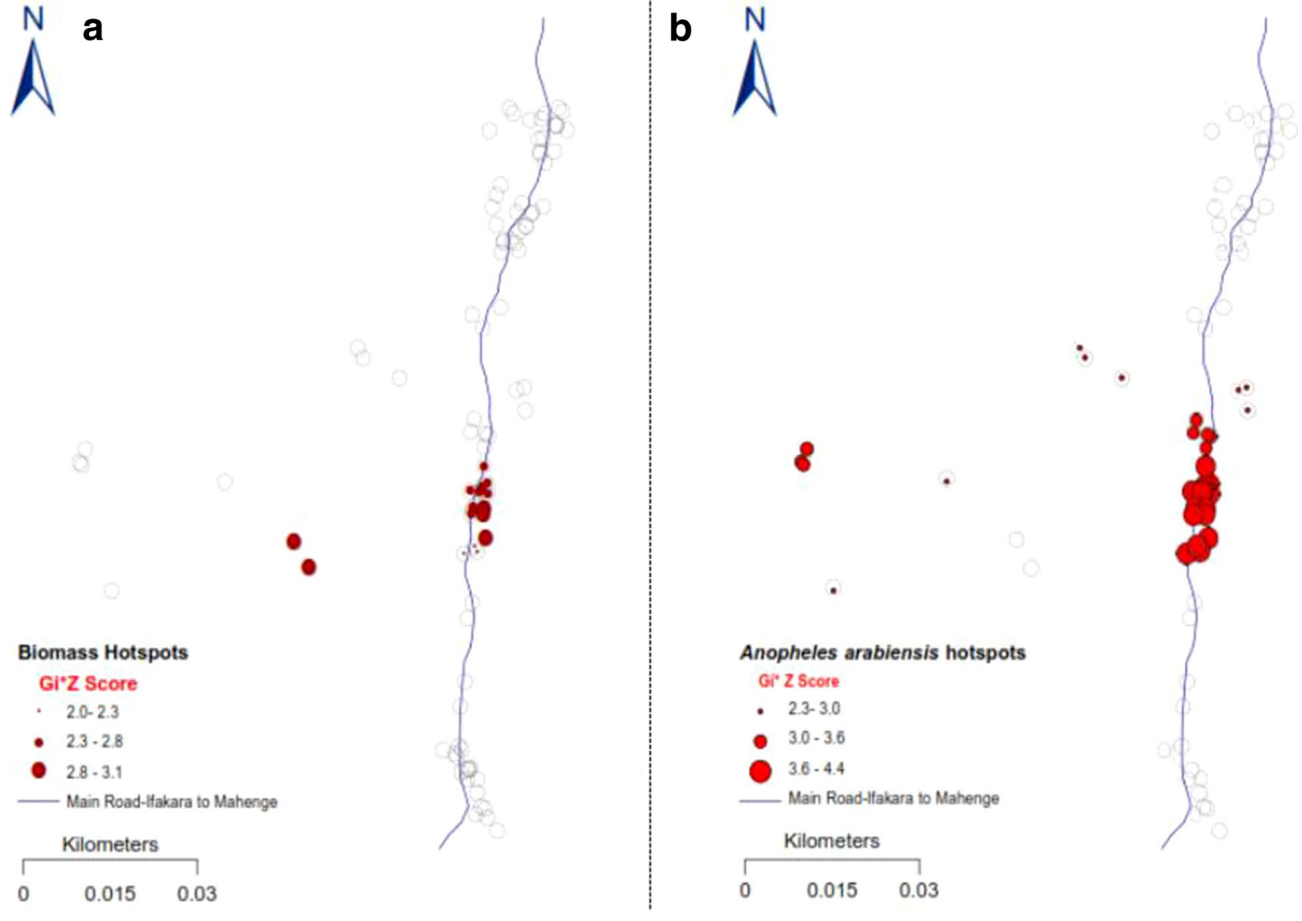
Maps showing statistically significant clusters of households with high occupancy (**a**) and statistically significant clusters of households with high densities of *Culex* mosquitoes in the same study area (**b**). The *grey circles* represent the rest of the households in the study area

There were clearly identifiable and statistically significant clusters of households with high densities of the two main malaria vectors, *An. arabiensis* and *An. funestus* in the central part of the study area (GI* Z ≥ 1.96, GI* P ≤ 0.005), but also significant clusters of *Culex* mosquitoes in the northern part of the study area (Figs. 4, 5, 6). Clusters of households with the highest occupancy occurred in these same geographical locations in the study area (GI* Z ≥ 1.96, GI* P ≤ 0.05). Since the study obtained the household occupancy data from all the houses where mosquito collections were also conducted, the analysis reveals that geographical clusters of households with the highest occupancy were individually the same clusters of households with highest densities of malaria vector species, but not *Culex* mosquitoes. Clustering of *An. arabiensis* (Fig. 4) and *An. funestus* (Fig. 5) were both geographically coincidental with clustering of household occupancy in the study area, but no such geo-coincidence was observed for *Culex* species, whose indoor densities were on the contrary geographically aligned to areas where houses with the lowest human occupancy were clustered (Fig. 6). The study found no statistically significant clusters of houses with high densities of *Mansonia* species mosquitoes in the study area.

### Species composition of malaria vectors and *Plasmodium* infection rates

A total of 39,754 *An*. *gambiae complex* and 14, 817 *An. funestus* group mosquitoes were assayed in the laboratory. All of the *An. gambiae s.l.* mosquitoes assayed were confirmed to be *An. arabiensis* (100 %), while 94 % of the mosquitoes from the *An. funestus* group assayed were *An. funestus s.s*. The rest of the *An. funestus* mosquitoes were *Anopheles rivulorum* (6 %). Overall, the sporozoite infection rates were 4.04 % in the *An. funestus* mosquitoes and 0.47 % in *An. arabiensis* during the study period.

## Discussion

Identification and targeting of high transmission foci particularly at fine scale levels within villages is essential for successful malaria control and eventual elimination [12, 14]. Transmission of malaria pathogens, like many other infectious agents, is heterogeneous over host populations but also over geographical space [21, 37], and this stratification increases significantly in reduced transmission settings [12, 14, 15]. Understanding these dynamics and how they are influenced by the various biotic and abiotic factors is essential to improving planning for interventions of ongoing malaria prevention strategies.

The study hypothesized that household occupancy (being proxy to household-level biomass), would influence not only indoor vector densities as shown in several previous studies [18, 19], but that it also influences mosquito dispersal within communities, and the resulting geographical distribution of human biting risk and pathogen transmission risks across these communities. By extension, it was assumed that overall directional movement of mosquitoes within villages is influenced by spatial distribution and demographic composition of households in these villages. As a result, locations where households with high biomass or occupancy are clustered would naturally form pockets of high transmission of mosquito-borne diseases, unless there are specific interventions or environmental variables, which significantly modulate such patterns.

Female mosquitoes need vertebrate host blood for reproduction and understanding this host-seeking behaviour would be essential for estimating the transmission of mosquito borne diseases, including malaria [17]. The host-seeking behaviour is influenced by many factors, including host odour cues, host density, dispersal ability of the mosquitoes and host distribution availability [17, 38]. Indeed, where distribution of human populations is heterogeneous, the distribution of adult mosquitoes also tends to be heterogeneous even if the breeding sites are uniformly distributed in the environment [21].

The controlled experimental hut studies verified earlier observations of correlations between vector densities and human biomass [18], but also provided a clear pattern of the seemingly linear relationships between these variables. The design of the experiment, using exit interception traps enabled mosquitoes freely—fly into huts and quantify the densities, by trapping them upon exit. The human volunteers participating in the study were fully randomly assigned to the huts on nightly basis, thereby excluding confounding effects of differential host attractiveness to mosquitoes [38]. Moreover, since the study restricted the age of volunteers to between 18 and 35 years, and relied on a fixed group of ten volunteers for this study, the observed associations between vector densities and volunteer numbers can be considered to represent correlations with human biomass. The experiment therefore provides the first of such datasets obtained under controlled environments in a malaria- endemic community, and lends itself to future use for fitting models that simulate mosquito host seeking and pathogen transmission.

Similarly, the field surveys also showed that houses with higher occupancy tended to have more mosquitoes as compared to houses with low occupancy, even though the indoor vector densities were also modulated by factors such as whether the eave spaces were open or not. In this study, the effects of trap-room and household occupancy were assessed by considering observed base-lines of at least one person per trap room versus at least two persons per household. This was because individual trapping rooms generally had at least one person while households generally had at least two members. Although the study observed several other household characteristics other than biomass and eave spaces, the analyses revealed that these were the two most influential variables on indoor vector densities in the study area. A study by Al-Eryani et al. in Yemen has also yielded similar evidence that the number of *An. arabiensis* was positively correlated with the number of occupants in the house [22].

There was a clearly observable geographical overlap in the spatial clustering of houses with high occupancy, and the clustering of houses with high densities of malaria vectors. The study analysed the data for household biomass separately from the data for vector densities, yet in both cases, there were significant clustering. The analysis thus provides a set of possible simple rules, which could be relied upon to predict at fine-scale, the parts of villages where the highest biting risk occurs and where intense, highly focalised vector control efforts would achieve greatest community-level impact. This study was conducted in an area which has historically had very high malaria transmission rates [3, 39], but where LLIN coverage is now evenly very high. Even then, this study suggests that by simply mapping household occupancy and their spatial distribution in the area, one would be able to rapidly identify places with the highest and lowest indoor vector densities, even without any vector trapping. This information is of major significance for spatial targeted interventions, particularly at fine-scale [20, 22], even within small administrative boundaries such as wards and villages.

Visual inspection of Figs. 4, 5 and 6 suggests that both intra- and inter-village variation in indoor mosquito-biting risk would be spatially correlated to household occupancy patterns, readily identifiable by using census and other demographic data in many other malaria-endemic countries. One point of caution is that whereas the assertions could hold true over geographically homogenous areas, and in the absence of any focalized vector control operations that disrupt mosquito-host seeking and density distributions such as IRS [40, 41] or larval source management [42], there are several other features, with potential to change or eliminate these spatial correlations. Features such as topography [43, 44], ground water and surface water flows [45], growth of urban centres and increased settlement densities [46], as well as agricultural cultivation [47], are examples that could disrupt the geographical coincidences observed. Indeed Thomas et al. recently concluded after analyses of data from The Gambia that mosquito dispersal would likely be landscape specific [44], necessitating that a reasonable level of characterization is conducted in the target communities. Despite these potential sources of discrepancies, the observations and experimental verifications have clearly determined that vector control operations at local district level could rely heavily on readily available household census data to predict basis risk patterns across villages, but that use of other data layers would improve the outcomes and overall predictions.

Since household-level analyses revealed increasing mosquito numbers with increasing number of occupants, the results of these geo-spatial analyses must be interpreted with caution. The increased community-level biting risk implied by these analyses is primarily because the increase in hazard levels, even if the individual level-exposure remained unchanged. Caution should be taken in the interpretation of these results so as not to imply that biting-risk per person was also increased inside household in the areas where host biomass was highest, even if the bite-related hazard was higher. Interpretations of the results should therefore be limited to the understanding that increased concentration of potentially infectious mosquitoes in these areas would enable more effective targeted control, with lower amounts of resources, and also that in such locations, even a low-level exposure, would result in significant risk of malaria infections. For example, it is likely to be more dangerous to sleep without a bed net in these locations with high concentrations of large households, than it is to sleep without a bed net in the rest of the villages. The results should therefore be examined from the perspective of community level protection from the increased biting risk. Since potentially infectious mosquitoes disperse towards, and eventually end up being most abundant in areas with highest household biomass concentrations, creating opportunities for targeted control of these vectors community-wide mass effect. Moreover, locations with clusters of large households can be considered as providing a significant level of protection to the smaller households elsewhere in the village [20], because mosquitoes are drawn mostly towards these locations, and away from the other areas. The greatest epidemiological value of the results is more on their potential as a way to target community-wide vector control and achieve mass effect on potentially infectious vectors, rather than as a way to predict individual risk.

Other than the experimental studies and community-wide vector surveys, this study also showed that the proportion of *Plasmodium*-infected *An. funestus* was far higher than proportions of *An. arabiensis* infected. The latter species thus plays a much greater role in malaria transmission, contributing up to 87.9 % of potential new infections in the study area, compared to only 12.1 % from *An. arabiensis*. No other infected *Anopheles* species was found during this study. The concern over the increasing role of local *An. funestus* populations remains an important one, given its greater competence as a vector of malaria. Although *An. arabiensis* is still the most prevalent of the vector species in the area, determining that ongoing residual malaria transmission is mostly mediated by *An. funestus* suggests that highly effective household-level interventions that target the indoor-feeding and indoor-resting behaviours of these vector species could still be highly applicable to bring down transmission levels. Such interventions would be greatly enhanced if spatially targeted to the parts of villages where host biomass is most concentrated. Studies by Lwetoijera et al. in southeastern Tanzania [48] and MacCann et al. in western Kenya [49] have also yielded similar evidence of increased role of *An. funestus*. The seemingly growing challenge would be further complicated in areas where the vector species is also increasingly resistant to insecticides commonly used for malaria prevention and control.

Considering both the experimental assays and the entomological survey data, this study indicates that household-level effects of host biomass on host-seeking and indoor vector densities are indeed transferable to community-level patterns. As a result, areas with concentrations of large households tend to have more mosquitoes than areas with sparsely distributed small households. Unfortunately, despite availability of vast quantities of household census and other demographic data regularly collected from large populations in many countries including Tanzania, no efforts have previously been made to triangulate such datasets with the knowledge of how vectors identify, locate and attack humans, so as to map the likelihood of mosquito-borne disease transmission within and between villages. One would propose that such triangulations should be considered as an initial step in the assessments of disease risk. The knowledge is essential for creating baseline estimates of transmission risk and disease burden, which enables actual transmission foci to be easily mapped on fine scales, using simply estimates of human biomass or household occupancy, from regular demographic surveys. In countries where HDSSs have been running for many years, such datasets could also be utilized to provide baseline spatial estimates for risk prediction and prioritization of interventions. Future studies may include modelling of malaria risk from existing datasets such as malaria indicator surveys (MIS) and Demographic Health Surveys (DHS), with the aim of confirming the results observed in this study. An obvious advantage here is that MIS and DHS datasets regularly record numbers and age of people in households and would provide reliable estimates of household-level biomass distribution across communities.

## Conclusion

Directly observable household level effects of household biomass on mosquito house entry are manifest at community-level spatial scales. These relationships result in a situation where areas with clusters of large households tend to have most biting mosquitoes, while areas with sparsely distributed small households have least biting risk. Overall, fine-scale and within-village clustering of indoor densities of major malaria vectors in this study area occurred in the same locations where houses with the greatest number of occupants were also clustered. It is hypothesized that in similar communities; the most intense foci of *Anopheles* biting risk could therefore be preliminarily predicted directly from household-level population census data. Where regular census data are available, with records of ages and numbers of people per household, it may be possible to rely on these maps to generate spatially defined high-resolution malaria risk maps, to support disease control programmes. However, since mosquito dispersal over space is often landscape-specific, it may also be necessary to identify what other factors significantly modulate these spatial relationships, and how these observations could be used to improve vector-borne disease mapping and control.
